# Characterization of Artifact Influence on the Classification of Glucose Time Series Using Sample Entropy Statistics

**DOI:** 10.3390/e20110871

**Published:** 2018-11-12

**Authors:** David Cuesta-Frau, Daniel Novák, Vacláv Burda, Antonio Molina-Picó, Borja Vargas, Milos Mraz, Petra Kavalkova, Marek Benes, Martin Haluzik

**Affiliations:** 1 Technological Institute of Informatics, Universitat Politècnica de València, Alcoi Campus, 03801 Alcoi, Spain; 2 Department of Cybernetics, Czech Technical University in Prague, 16000 Prague, Czech Republic; 3 Internal Medicine Department, Teaching Hospital of Móstoles, 28935 Madrid, Spain; 4 Department of Diabetes, Diabetes Centre, Institute for Clinical and Experimental Medicine, 14021 Prague, Czech Republic; 5 Department of Medical Biochemistry and Laboratory Diagnostics, General University Hospital, Charles University in Prague 1st Faculty of Medicine, 12108 Prague, Czech Republic; 6 Hepatogastroenterology Department, Transplant centre, Institute for Clinical and Experimental Medicine, 14021 Prague, Czech Republic; 7 Obesitology Department, Institute of Endocrinology, 11694 Prague, Czech Republic; 8 Experimental Medicine Centre, Institute for Clinical and Experimental Medicine, 14021 Prague, Czech Republic

**Keywords:** sample entropy, fuzzy entropy, blood glucose, signal classification

## Abstract

This paper analyses the performance of SampEn and one of its derivatives, Fuzzy Entropy (FuzzyEn), in the context of artifacted blood glucose time series classification. This is a difficult and practically unexplored framework, where the availability of more sensitive and reliable measures could be of great clinical impact. Although the advent of new blood glucose monitoring technologies may reduce the incidence of the problems stated above, incorrect device or sensor manipulation, patient adherence, sensor detachment, time constraints, adoption barriers or affordability can still result in relatively short and artifacted records, as the ones analyzed in this paper or in other similar works. This study is aimed at characterizing the changes induced by such artifacts, enabling the arrangement of countermeasures in advance when possible. Despite the presence of these disturbances, results demonstrate that SampEn and FuzzyEn are sufficiently robust to achieve a significant classification performance, using records obtained from patients with duodenal-jejunal exclusion. The classification results, in terms of area under the ROC of up to 0.9, with several tests yielding AUC values also greater than 0.8, and in terms of a leave-one-out average classification accuracy of 80%, confirm the potential of these measures in this context despite the presence of artifacts, with SampEn having slightly better performance than FuzzyEn.

## 1. Introduction

The main idea of diabetes control is assessing a time series (blood glucose) [1]. Traditionally, this was performed by means of punctual fasting blood measurements. This was obviously inappropriate. A slight improvement was self-monitoring through serial capillary blood controls performed by the patient [2]. However, again, this was cumbersome and offered a poor overview of the time series. An important step was the use of glycosylated Hemoglobin (HbA1c), which provided an integrated assessment of the glucose blood levels of the last 8–12 weeks [3]. This became a standard of care for many years. However, HbA1c has several drawbacks (it depends on hemoglobin levels, on the life span of red blood cells, etc.) [4]. Furthermore, it is increasingly clear that raw blood glucose level is not the only relevant variable and that the glycemic profile (complexity, variability), which is not evaluated by HbA1c, is also clinically significant [5]. Therefore, the development of a Continuous Glucose Monitoring System (CGMS) was universally perceived as an important improvement. Although its cost and some technical problems are refraining its generalization, many authors believe that CGMS will soon become a standard of care [6].

The analysis of glucose time series using entropy estimators is a very difficult endeavor. Because of the technical constraints and patient inconvenience during portable CGM, records are usually very short (24 or 48 h, non-uniformly sampled at 5-, 10- or 20-min intervals, usually). As a result, the length of the time series available falls well below the recommended minimum length of $10^{m}$, where *m* is the embedded dimension used in entropy estimators derived from Approximate Entropy (ApEn) [7], such as Sample Entropy (SampEn) [8] or Fuzzy Entropy (FuzzyEn) [9], with usual values of m=2,3, or even higher.

In addition, devices of CGM need to be recalibrated, up to several times per day [10], and mainly at the beginning of the ambulatory monitoring. Missing values are also frequently found in these records, due to device or sensor errors [11]. Other problems encountered in blood glucose time series are sensor out-of-range or sensor disconnection values, causing a signal saturation or a flat reading during a time interval usually involving several contiguous samples.

Although the advent of new blood glucose monitoring technologies may reduce the incidence of the problems stated above, incorrect device or sensor manipulation, patient adherence, sensor detachment, time constraints, adoption barriers or affordability [12,13,14,15,16] can still result in relatively short and artifacted records, as the ones analyzed in this paper.

CGM provides essential information related to the diagnosis and prognosis of the subjects. Moreover, CGM will become an indispensable tool for closed-loop control of future artificial pancreas, and the processing of any relevant information must be fast, robust and reliable. Traditionally, besides the usual all-or-nothing thresholds applied to detect hyper- or hypo-glycemia [17,18], this processing has been mainly based on a set of glucose variability metrics [19,20,21]. More recently, this variability analysis has evolved into a more elaborated scheme using time series regularity, complexity or entropy estimators [22]. Among all these proposed estimators, Detrended Fluctuation Analysis (DFA) [23] is probably the most used method in the context of glucose data. This measure quantifies the long-term correlations of non-stationary time series, and it has been used to assess the fluctuations of glucose in normal daily life in diabetic and non-diabetic subjects [24], as a predictor for the development of diabetes of patients at risk [25], to study the initial phases of glucose metabolism dysfunction in hypertensive patients [26], as a marker of risk for critical patients [27] or to estimate insulin resistance also in diabetic and non-diabetic subjects [28].

Other entropy estimators based on ApEn have been less extensively used in this context [29], probably due to the problems stated above. Glycemic ApEn has been correlated with patient outcome after surgery, among other metrics [30]. SampEn, its derivative multiscale entropy, mainly, has been used to study the complexity of glucose dynamics in diabetes [31]. So far, FuzzyEn has not been included in any research work related to the analysis of blood glucose time series. However, these two methods have been extensively and successfully used in many other time series classification studies [9,32,33,34,35,36,37,38], not only physiological records [39,40], and have also demonstrated their robustness against signal artifacts [41,42,43]. This is why these two entropy statistics were chosen for the present study.

Given the claimed better performance of FuzzyEn reported in the literature [9], in comparison with its predecessors ApEn and SampEn, we wanted to study the applicability of this new metric to glycemia data, taking into account the possible ill effects caused by the record features stated above, and their characterization. Based on the dataset obtained from a study of the endocrine consequences of duodenal-jejunal exclusion [44], this paper comparatively assesses the capability of SampEn and its derivative FuzzyEn to distinguish between classes, under different conditions in terms of record length, artifacts and border effects. The clinical implications of such a classifier can be varied and diverse. Changes in glucose dynamics could be correlated with other anthropometric, biochemical or hormonal characteristics [44,45] in order to try to anticipate the rate and intensity of metabolic improvements after the exclusion and better understand the possible mechanisms of its effects. It could also be used as a screening tool for patient/treatment selection.

The performance was assessed using the Area Under the ROC Curve (AUC) [46] values obtained for the classification of two input classes (Months 1 and 10 for the database described in Section 2.2). AUC is a widely-used measure in a diversity of classification schemes, including those based on entropy metrics in the context of biomedical applications [32,47,48,49,50,51].

**Figure 1:**
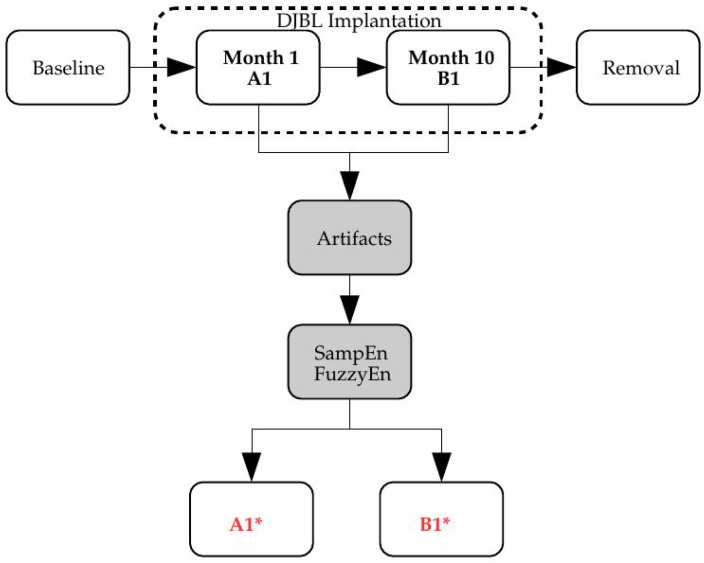
Block diagram. The Duodenal-Jejunal Bypass Liner (DJBL) was implanted into a number of obese patients with type 2 diabetes mellitus. Glucose time series for each one were recorded one month and 10 months after implantation. This study analyses the possible differences in the glucose control at those stages and the influence that artifacts found in these records may exert on non-linear metrics performance, specifically SampEn and FuzzyEn. The outcome of the process is the estimated classes $A1^{*}$ and $B1^{*}$.

The metric for the classification was SampEn or FuzzyEn, and the input time series underwent different transformations to account for the effects targeted in this characterization study: record length (Section 3.2), missing samples (Section 3.3), sensor saturation (Section 3.4) and time offset (Section 3.5). The block diagram of the analysis proposed is shown in Figure 1.

## 2. Materials and Methods

### 2.1. SampEn and FuzzyEn

SampEn was first introduced in [8], as an improvement of ApEn, and FuzzyEn in [9], also as an enhancement of SampEn. These methods were devised to characterize the level of irregularity, complexity, randomness or predictability found in time series, which is related to the dynamics of many physiological systems [52], as is the case for the gluco-regulatory system. Both algorithms are quite similar, but FuzzyEn replaces the dissimilarity measure by a fuzzy function (membership function); and the subsequences are normalized in terms of zero mean, before computing such dissimilarity.

The input to both methods is a time series x of length *N*: $x=\left\{x_{1},x_{2},x_{3},\cdots ,x_{N}\right\}$, from which a set of ordered subsequences $x_{i}$ of length m<<N is extracted: $x_{i}=\left\{x_{i},x_{i+1},x_{i+2},\ldots ,x_{i+m-1}\right\}$.

In SampEn, the maximum distance between 2 different subsequences $d_{ij}=\max(|x_{i+k}-x_{j+k}\left|\right),0\leq k\leq m-1,j\neq i$ is computed. This distance is thresholded using a predefined parameter *r*, the number of distances found, ∀j, and for a specific *i*, within such a threshold, stored in a counter $B_{i}\left(r\right)$. This process is repeated ∀i and the final value averaged:

$$B^{m}\left(r\right)=\frac{1}{N-m}\sum_{i=1}^{N-m}B_{i}^{m}\left(r\right)$$

An additional counter $A^{m}\left(r\right)$ is obtained using m⟶m+1. Finally, SampEn is obtained as:

(1) $$\mathrm{SampEn}\left(m,r,N\right)=-\log\left[\frac{A^{m}\left(r\right)}{B^{m}\left(r\right)}\right]$$

In FuzzyEn, each local mean is first subtracted from every subsequence: $y_{i}=\left\{x_{i}-\mathrm{mean}\left(x_{i}\right),x_{i+1}-\mathrm{mean}\left(x_{i}\right),x_{i+2}-\mathrm{mean}\left(x_{i}\right)\ldots ,x_{i+m-1}-\mathrm{mean}\left(x_{i}\right)\right\}$, i=1,…,N−m+1. The dissimilarity is now computed as $D_{ij}=\mu \left(d_{ij},r\right),0\leq k\leq m-1,j\neq i$, where μ is the fuzzy function selected, usually the exponential function $\exp(-{\left(d_{ij}/r\right)}^{n})$. The counters now become:

$$\phi_{i}^{m}\left(r\right)=\frac{1}{N-m-1}\sum_{j=1,j\neq i}^{N-m}D_{ij}^{m}$$

$$\varphi^{m}\left(r\right)=\frac{1}{N-m}\sum_{i=1}^{N-m}\phi_{i}^{m}\left(r\right)$$

Finally, FuzzyEn is obtained as:

(2) $$\mathrm{FuzzyEn}\left(m,r,n,N\right)=\left[\log\varphi^{m}\left(r\right)-\log\varphi^{m+1}\left(r\right)\right]$$

The performance of both metrics depends on the value of the parameters *m* and *r*, and specifically for FuzzyEn, *n*. These values are very application specific, and for optimal performance, an exploratory analysis of a range of values must be carried out in advance (grid search). During the experiments, and in all cases, *r* values tested ranged from 0.15–0.30, in 0.01 steps, and *m* values from 1 up to 3. This way, the influence of the parameters on the results was minimized, and only optimal configurations in the specified subset, in terms of maximum AUC, and in accordance with the recommended values for *m* and *r*, were considered.

### 2.2. Experimental Dataset

The experimental dataset was recorded at the Third Department of Medicine, Department of Endocrinology and Metabolism, Charles University in Prague, Czech Republic. This database contains 91 records of 30 diabetic patients that underwent a duodenal-jejunal bypass liner implantation. Records contain measurements at baseline (before implantation), 1 month and 10 months later and 3 months after removal. Durations span from a few hours (796 samples being the shortest) up to more than 6 days for a few records (2022 samples being the longest), with a sampling period of 5 min. Sensors had to be recalibrated twice per day, mainly at the beginning of the recordings, and that is why possible border effects were likely to be present during the first hours or even days of the recordings due to the learning curve.

**Figure 2:**
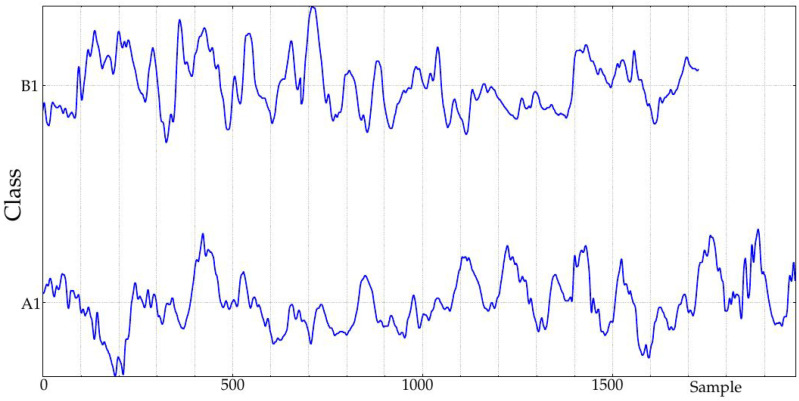
Example of two signals of the experimental database. Classes A1 and B1 correspond to records obtained after one month and 10 months of duodenal-jejunal bypass liner implantation, respectively. The sampling period was 5 min.

In this study, only records obtained during the implantation (at Month 1, class A1, 24 records, and at Month 10, class B1, 23 records, 47 in total) were studied (Figure 2). The rationale of this selection was to study the possible effects of such implantation. In the seminal endocrine study [44], many physiological characteristics exhibited significant differences that could be arguably translated into measurable glucose control changes from A1 to B1. Specifically, that study assessed the influence of The Duodenal-Jejunal Bypass Liner (DJBL) on anthropometric parameters, glucose regulation and the metabolic and hormonal profile of diabetic obese patients. All the subjects experienced a significant body weight, waist circumference and body fat reduction, starting at one month after implantation, which further progressed until the 10 month. Glucose variability decreased during the period from the first month until the 10-month follow-up, which can be related to changes in glucose complexity or dynamics, as studied here [52]. This effect was lost after DJBL removal, and that is why only classes A1 and B1 are analyzed in the present study. Fasting plasma insulin and C-peptide concentrations also decreased during that period. Other changes can be checked in [44]. Of all 47 records, 36 corresponded to the same subjects (18 in each class). Incomplete pairs were therefore discarded. Additionally, paired tests always need less subjects [53]. The percentage of missing samples in these records was close to 10% in the worst case, with a few records with no missing samples at all. Further details of this database can be found in [44].

## 3. Experiments and Results

### 3.1. Parameter Optimization

**Figure 3:**
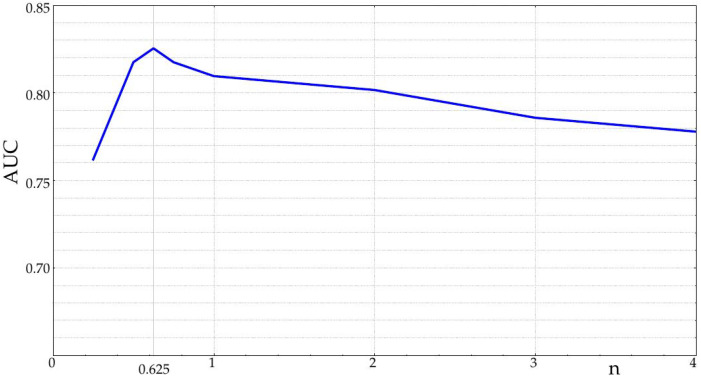
Optimization of the FuzzyEn *n* parameter for the experimental database. The plot starts at n=0.25. The highest AUC (0.82) was obtained for n=0.625. At n=2, the dissimilarity function becomes Gaussian.

The parameters *m* and *r* for SampEn and FuzzyEn were optimized using a grid search, namely AUC was computed for all their values proposed in Section 2.1, and the optimal configuration was taken as that for which AUC was maximal. For practical purposes, to keep the computational burden within reasonable limits, the *n* FuzzyEn parameter was optimized for a single case instead, six day-long records, taken at the center of the records. The result of the optimization of the *n* parameter was n=0.625, with an AUC = 0.82 (Figure 3).

For illustrative purposes, Table 1 shows the optimal configuration achieved for records of six days, best cases, to provide a more complete picture of the performance of the classification. A column is included to depict the statistical significance of the results in terms of a Mann–Whitney test, since the results were not normally distributed.

**Figure 4:**
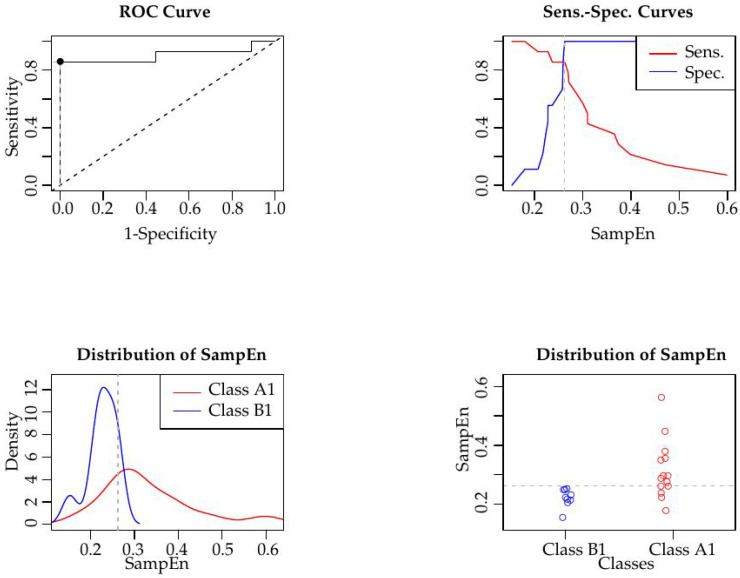
ROC analysis of the results in Table 2.

The numerical results are shown in Table 2 for the case SampEn(1,0.17,1728). This configuration achieved a specificity of 100% (correctly classified B1 records, with a 95% confidence interval of [0.629,1]), sensitivity of 85.7% (correctly classified A1 records, with a confidence interval of [0.562,0.975]), and a global classification accuracy of 91.3%, with an optimal threshold of 0.2625. These numerical results are graphically depicted in the ROC analysis [54] of Figure 4. With a parametric ROC fitting [53], the AUC is 0.82, with an asymptotic confidence interval of [0.627,1] and p=0.0012. With a nonparametric ROC fitting (empiric, top-left plot in Figure 4), AUC is 0.90, with a confidence interval [55] of [0.766,1], with $p=1.08\times 10^{-8}$, and a standard error estimation of 0.0708. The sample size that required estimation using [53], with power = 0.8 and AUC = 0.8, was 20, 10 for each class, which is close to the actual size in this study, 23 (14 and 9).

The maximum AUC was achieved for m=1 and r=0.17 for records of six6 days long, in general for *r* values close to 0.2, and for m=1 for SampEn and m=3 for FuzzyEn, in accordance with the general guidelines for these parameters. In addition, for the best case, a Leave-One-Out (LOO) validation procedure was applied to further assess the validity of this performance. Results for 25 tests with replacement are shown in Table 3.

Since the algorithm differences between FuzzyEn and SampEn are the dissimilarity function and the epoch mean normalization, in order to find out what was the feature that played the major role in the result differences, the optimization procedure was repeated without the epoch mean normalization. The new results with the modified FuzzyEn method, FuzzyEn*, are shown in Table 4.

According to the results in Table 4, FuzzyEn performance does not significantly vary if the epochs are not normalized in terms of the mean. Therefore, the other algorithm difference, the dissimilarity function, seemed to play the main role in the lower performance of FuzzyEn in this case.

### 3.2. Influence of Record Length

The influence of record length is a very well-known weakness of most entropy metrics, not only those studied here. In contexts where the acquisition stage ensures a sufficiently long time series, this weakness can be easily overcome. However, this is not the case with blood glucose time series, where the invasiveness of the sensors and the limitations of the process (low sampling rate, battery life, calibration) do not enable measurements longer than a few days at most.

**Figure 5:**
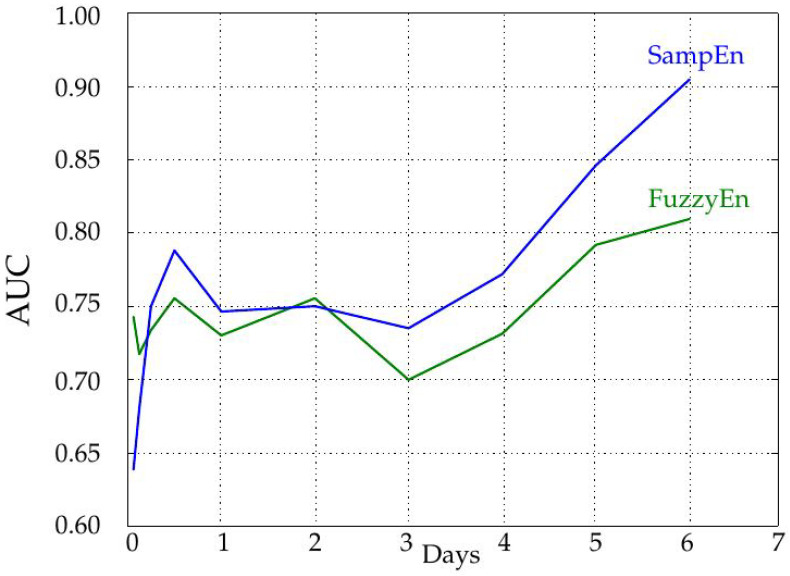
Influence of record length. As the duration of the time series increases, the classification performance in terms of AUC also increases significantly, mainly from Day 4. The results for FuzzyEn are lower than those of SampEn. This analysis was performed using the parameter values m=1 and r=0.17, optimal for the six day-long series, but suboptimal for shorter lengths (Table 1). This may explain the significant performance increase at five and six days, along with the availability of a number of samples beyond 1000. In a real situation, it is more usual to find out the best parameter configuration for the most important case and to use it for all the cases. For a length performance comparison using locally-optimized parameters, see Table 1.

This experiment was carried out using the six day-long records of the database. The length of the records was progressively increased from 1–6 days in 288 samples steps (one day, taken at the center of the complete record to avoid border effects), and the parameters were optimized as described in the previous section. The AUC for each length was computed using both estimators. The results are shown in Figure 5.

**Figure 6:**
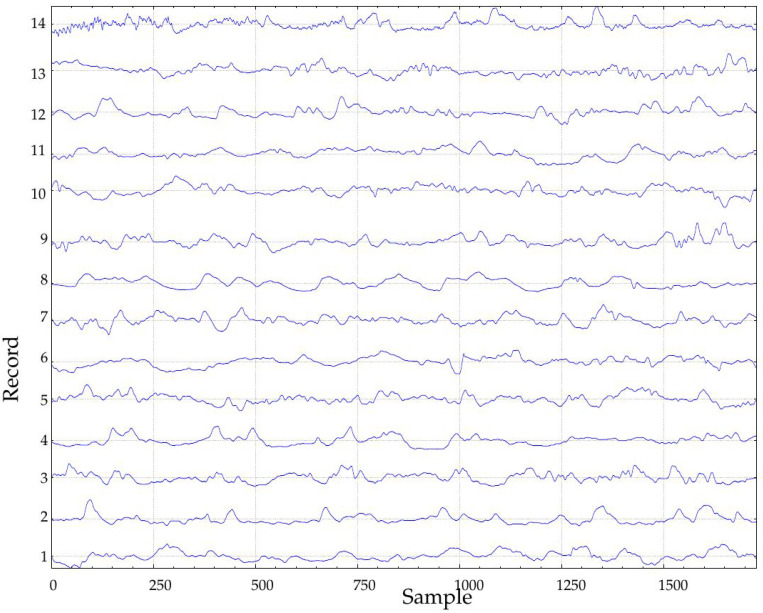
Records of class A1 of length at least six days used in the experiments.

**Figure 7:**
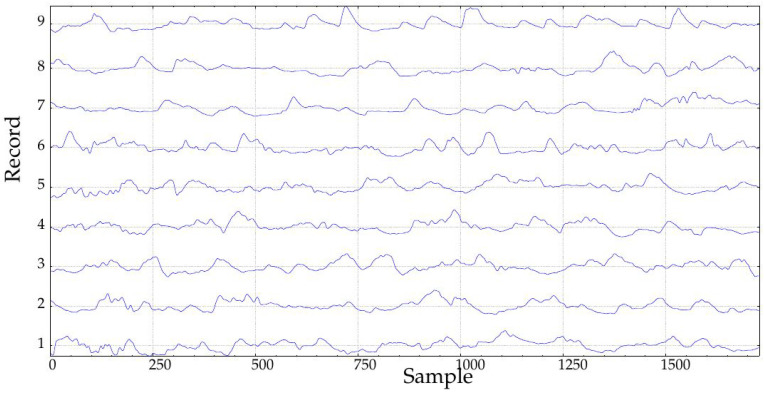
Records of class B1 of length at least six days used in the experiments.

Since most records were at least 288 samples long, but only 23 out of 47 achieved the required six-day duration, only those 23 were used in the experiments for all lengths (Figure 6 and Figure 7). This way, the final dataset remained constant during the computation of all the tests. For a single day, namely the mid-288 samples, lengths of 18, 36, 72 and 144 samples were also studied. The maximum performance was achieved for the longest records, as expected. At Day 6, AUC using SampEn was 0.90 and 0.82 for FuzzyEn. For durations shorter than four days, both metrics yielded approximately the same results (AUC close to 0.75).

**Figure 8:**
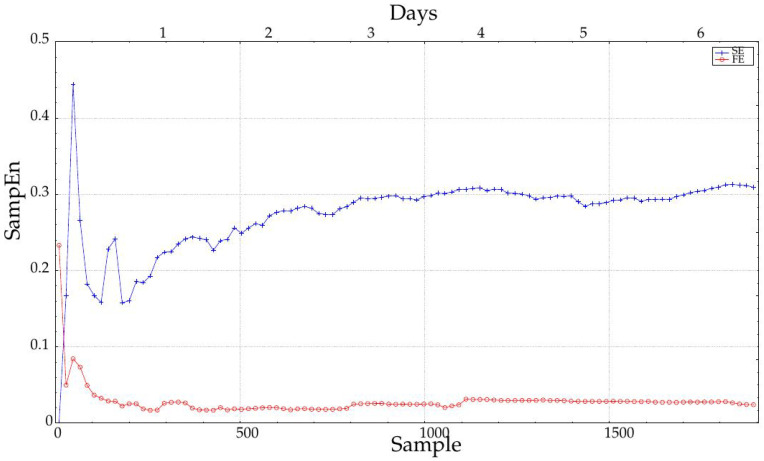
Sensitivity of SampEn and FuzzyEn to record length. For records shorter than 1000 samples (3.5 days; see the scale at the top axis), SampEn is quite unstable. FuzzyEn is less sensitive to record length, although it becomes really stable at 1100 samples. Values shown correspond to the averages of all the records six days long.

It is important to note that most entropy measures are very sensitive to input record length, and this fact may contribute to the differences in performance with the six day-long records and their shorter counterparts. Namely, the differences found are not only due to physiological or monitoring reasons, but also due to analytical needs. This sensitivity is graphically illustrated in Figure 8. SampEn becomes stable at approximately 1000 samples, nearly four days, whereas FuzzyEn stability is reached at 200–300 samples, one day. This may also explain why the performance of FuzzyEn does not vary with length as much as that of SampEn, mainly from Day 4, as depicted in Figure 5.

### 3.3. Influence of Missing Samples

**Figure 9:**
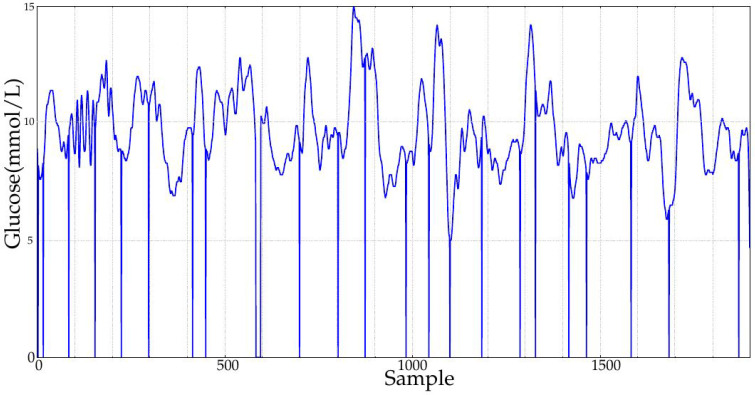
Real record with missing samples. The values not found are considered as zero for representation. This record exhibits a missing sample rate slightly higher than 10%.

Missing samples is a common issue in blood glucose time series (Figure 9), but there is no study so far that has characterized the impact of the incomplete data on the signal classification performance. Researchers usually discard records or epochs with too many missing samples (20% is a heuristic threshold we had used in the past), and a common approach for short series of missing data is to reconstruct those values from the neighboring observations available.

The experimental database included many records with missing samples, but they were interpolated before the experiments (pre-filtering). The assessment of the impact of missing samples was conducted using random synthetic ones (spikes down to zero), with customized percentages ranging from 2.5% up to 10%, in 2.5 steps. The results are shown in Table 5.

In order to gain more insight into the influence of missing samples, the 10% case in Table 5 was re-analyzed after applying an interpolation scheme to remove all the gaps. Using the fuzzy metric, from an AUC of 0.74, sensitivity of 0.72±0.075 and specificity of 0.686±0.084, the performance after interpolation was back to that of the initial case shown in Table 1. This was also the case for SampEn, which suggests that linear interpolation is a suitable tool to account for missing samples.

Although more than one missing sample can be found consecutively (missing epochs), that case is easily detected and usually addressed splitting the record at that point (the longer the gap, the less reliable the calculations become [11]), and therefore, only the most frequent case of a single missing sample was analyzed.

### 3.4. Influence of Sensor Saturation

**Figure 10:**
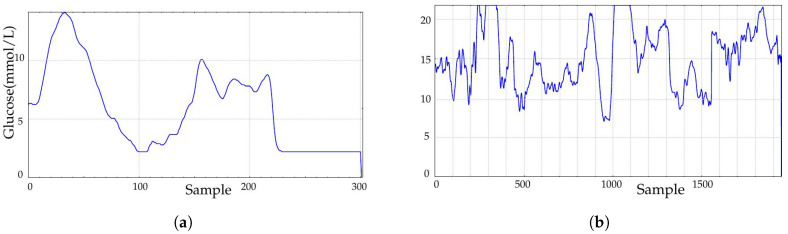
Influence of sensor saturation. Records remain flat for some samples at one or both ends of the device measurement scale. (**a**) Example of saturation at low values. Data at Locations 100 and 225 include some continuous readings at 2.2 mmol/L, mainly at the record end. (**b**) Example of saturation at high values. Data at Locations 300 and 1000 include some continuous readings at 22.2 mmol/L.

Sensor saturation refers to the impossibility for the device to provide readings above (end of scale) or below (minimum sensitivity) some certain thresholds, usually due to technical constraints or sensor attachment problems. This is very usual in real continuous blood glucose monitoring. Before the user or the physician notices there is something wrong with the placement of the sensor or any other device malfunction, such as the loss of or incorrect calibration parameters, some samples have already been acquired. Figure 10a depicts a record with low saturation values at 2.2 mmol/L, whereas in Figure 10b, the record is saturated at 22.2 mmol/L at some points (limitation of the recorder used). These records did not belong to the experimental dataset.

The assessment of the impact of saturation was conducted using random saturation pulses, of 22.2 mmol/L of amplitude, and with customized lengths of six up to 60 samples, in six-sample steps. They were randomly located in the records in a similar way as the spikes, but only one pulse per record. The quantitative results are shown in Table 6.

### 3.5. Influence of Time Offset

Glucose time series are not stationary [56]. From a practical perspective in this study, this means the values obtained for the entropy metrics using a certain time window will surely differ from those obtained at another time window. In addition, many doctors have reported intuitively that the beginning of the records could probably be less reliable or stable. Clinically, there are also temporal changes due to initial calibrations of the measuring devices, the learning curve related to their proper use or wearing and changes in treatment adherence before or after a clinic appointment [57]. This is known as the white-coat effect, and it is a well-known disturbance that causes significant temporal changes in physiological markers during clinical visits [58,59]. These changes can arguably play a role in the correct analysis of the resulting time series, since they are not related to the dynamics of the glucose control systems, but to other irrelevant external factors.

Therefore, the entropy-likely fluctuations associated with temporal changes had to be studied with regard to the signal classification capability of these methods. For this purpose, AUC was computed for three-day time windows shifted one day from zero up to three days, with two days overlapping, using records of six days long. The results of these experiments are shown in Table 7, including a statistical significance analysis.

## 4. Discussion

This study analyses the impact of the typical artifacts found in blood glucose records on the class segmentation capabilities of SampEn and its derivative FuzzyEn. The influence of the parameters is practically removed by a grid search of the optimal configuration for the purpose of each experiment.

The main metric to quantify the performance of the methods was AUC, including a statistical significance assessment for some cases, an LOO cross-validation, and a global classification accuracy score for the optimal configuration. AUC is a very popular metric to assess the performance of a classifier due to its simplicity, robustness (insensitive to class asymmetry) and straightforward interpretability: if a classifier A has a greater AUC than a classifier B, A has a better average performance than B [46]. AUC quantifies the classifier’s ability to avoid false classification [60], with a performance threshold for random guessing of 0.5. In other words, the closer AUC is to 1.0, the better is the expected performance of the classifier.

The influence of the record length was characterized by increasing the number of samples used in the entropy calculations in steps of 288 samples, that is one day. As depicted in Figure 5, the longer the record, the higher the AUC, and therefore, the more separable the two classes are. The AUC remains more or less constant up to four days, and then, it increases significantly. This could be due to the achievement of a length that enables a more robust entropy estimation (greater than 1000 samples), as recommended in some works [7] and visually justified in Figure 8. However, it does not mean SampEn or FuzzyEn are not usable at short lengths, because the important feature is the dissimilarity between entropy values from each class, not their absolute values. In any case, SampEn yields better results than FuzzyEn for all the lengths except for extremely short records (only 18 or 36 samples). It is important also to note that records were cut from data at the center of the entire available records to avoid possible border effects and ensure more data stability.

The presence of one-sample gaps in the time series did have a significant impact on the separability of the two classes under analysis. Both metrics worsened their performance at each step, although FuzzyEn appeared to be a little bit more robust. Arguably, it can be hypothesized that these missing samples may hinder the classification of blood glucose records, and they should be avoided, if possible, or filtered out with some kind of interpolation. For real interference levels of 10%, the separability becomes very poor, even for a baseline AUC higher than 0.90. It is also important to note that this analysis was carried out in terms of classification performance, not in terms of changes in absolute entropy values, which surely took place [43].

Sensor saturation is another record disturbance that also significantly damages classification performance. Even for very short saturated epochs (60 samples, five hours at one sample per five minutes, 3.5% of the six-day records), the two classes become almost undistinguishable. This is also another quite frequent issue in most blood glucose records, and it is almost impossible to remove using signal processing techniques, since the real signal cannot be reconstructed. Therefore, this disturbance should be detected and corrected as soon as possible at the acquisition stage.

The possible effect of the specific time window on the analysis is quantified in Table 7. Time windows of three days were taken from the beginning of the records of at least six days long of the experimental database, and the calculations of SampEn and FuzzyEn were repeated for all the possible windows, shifted one day in each case, with two days overlapping. This experiment was devised to find out if the global differences found were due only to non-stationary changes, or if the differences were regularly distributed along the entire records. Although class differences are fairly significant in most of the epochs analyzed, there is a clear trend towards higher differences at later stages. This may be due to a more stable glucose monitoring, a better device calibration or just a correlation with the learning curve linked to the whole process of CGM. It is important to note, however, that the beginning of the records seems to be the most unreliable part in terms of class segmentation, namely there seems to be a border effect on CGM records that should be avoided during analysis.

In all cases analyzed, the class separability was higher using SampEn. Even skipping the subsequence mean normalization stage in FuzzyEn, the SampEn performance was higher, which means that this type of record requires a sharp dissimilarity function. The input parameters were quite consistent and stable. As for *m* and *r*, there was small intra-class variability, with optimal values close to m=1 for SampEn, m=3 for FuzzyEn and r=0.2 for both. The optimal *n* obtained for FuzzyEn was very low, n=0.625.

## 5. Conclusions

There are no standardized metrics for CGMS evaluation and perhaps different goals on different patients may require different metrics. Specifically concerning complexity, it is crucial to choose the right complexity metric, optimize its parameters and analyze the influence of sample length, missing data, sensor saturation and time offset. This is precisely the goal of the present paper. We assessed the metric’s discriminating power comparing two time series of a sample of patients before and 10 months after undergoing a therapeutic maneuver (DJLB) known to modify glucose metabolism, and we evaluated if and how these metrics were able to detect those changes.

CGM data are an extremely useful source of metabolic information with a myriad of current and future applications. However, records are usually very short and noisy, mainly in terms of missing samples and sensor saturation, and these artifacts may arguably interfere with the correct interpretation of the results using the otherwise successful entropy features. This study was aimed at characterizing the changes induced by such artifacts, enabling the arrangement of countermeasures in advance.

As expected, record length is pivotal for a reliable entropy assessment of the records. Although classification potential was always higher than 0.75, even for 288 samples, measured in terms of AUC, more robust results were obtained for longer records. In any case, we would recommend not to use records shorter than 24 h, since it is important to cancel out the chronobiological effects on the glucose dynamics, for example sleep and the fasting periods and meals during the day. For shorter series, it would be necessary first to characterize these chronobiological effects.

Missing samples seem to interfere significantly with the estimation of the underlying dynamics in glucose time series. Even with a very low ratio of 2.5%, there was a significant reduction of the AUC obtained, and this reduction was consistent along all the ratios tested. As for relatively usual higher ratios of 10% missing samples, it could become impossible to distinguish between the two classes. Fortunately, this artifact can be easily removed by just interpolating the missing samples, and this should be a routine procedure in the preprocessing stages of this kind of biomedical record.

The saturation of readings is also a usual disturbance in CGM data. A single epoch of six saturated consecutive values in the entire 1728 sample record has a great impact on the AUC, greater than that of missing samples. Moreover, this artifact is very difficult to remove since it would entail the reconstruction of the missing values. As a consequence, it would be advisable to implement some kind of alarm to detect this situation and implement corrective measures as soon as possible, during the acquisition stage.

Time offset is another key element to ensure proper interpretation of the class separability. The beginning of the records is less powerful in this regard, whereas the maximum separability is achieved at later stages. It is well known that initial calibration and sensor stabilization may cause border effects in these recordings, but more recordings, including timestamps for events, would be necessary to find out exactly what are the factors influencing this trend. As a general recommendation, the longer the records, the better, and if possible, discard the initial samples for analysis.

As for the statistics employed, SampEn outperforms FuzzyEn in all cases. Despite being an evolution and improvement, FuzzyEn does not achieve the AUC obtained with SampEn, in contrast with previous works [42], where FuzzyEn was clearly better. This means that derivatives are not necessarily always more effective than the original metrics, and more characterization studies would be necessary to define the optimal application domains in each case. However, despite the limitations of CGM data, classical regularity estimators can successfully be applied, as with other biomedical records.

## Figures and Tables

**Table 1:** Results of the parameter optimization process, using SampEn and FuzzyEn. Cases shown correspond to the best cases among all the cases tested, ranked by AUC result. Significance is confirmed by sensitivity, specificity and the *p*-value of the Mann—Whitney test.

Metric	*m*	*r*	*n*	*N*	Records	AUC	Sensitivity	Specificity	*p*
SampEn	1	0.17	NA	1728	23	0.90	1	0.86	0.0015
SampEn	2	0.17	NA	1728	23	0.88	1	0.78	0.0022
SampEn	3	0.17	NA	1728	23	0.87	0.89	0.78	0.0034
SampEn	1	0.20	NA	1440	30	0.84	0.92	0.65	0.0033
SampEn	1	0.20	NA	1152	36	0.78	0.87	0.65	0.0053
SampEn	1	0.20	NA	864	44	0.73	0.85	0.56	0.0096
SampEn	1	0.17	NA	Any	47	0.70	0.65	0.71	0.0213
SampEn	3	0.17	NA	Any	47	0.70	0.70	0.67	0.0206
SampEn	3	0.15	NA	Any	47	0.69	0.73	0.66	0.0254
FuzzyEn	3	0.24	0.625	1728	23	0.82	0.88	0.71	0.0151
FuzzyEn	3	0.15	1.0	1728	23	0.81	0.77	0.78	0.0183
FuzzyEn	3	0.27	0.625	1440	30	0.78	0.76	0.70	0.0178
FuzzyEn	3	0.11	0.625	1152	36	0.73	0.69	0.70	0.0226
FuzzyEn	2	0.12	0.625	864	44	0.69	0.66	0.61	0.0385
FuzzyEn	3	0.24	0.625	Any	47	0.61	0.61	0.67	0.1870

**Table 2:** Numerical SampEn results for the 6 day-long records of both classes.

	Class A1	Class B1
1	0.3098	0.2577
2	0.2625	0.1529
3	0.2280	0.2367
4	0.3652	0.2597
5	0.2713	0.2174
6	0.3099	0.2222
7	0.2998	0.2278
8	0.4708	0.2072
9	0.2694	0.2585
10	0.5985	–
11	0.1798	–
12	0.2859	–
13	0.3738	–
14	0.3985	–
Global	0.3303±0.1067	0.2267±0.0337

**Table 3:** LOO results. One record of each class was randomly omitted from the dataset, and the AUC analysis was carried out on the remaining (training) data. A classification threshold was then computed and later applied to the omitted (validation) records.

Record A1	Record B1	AUC	Threshold	SampEn Record A1	SampEn Record B1	Accuracy
7	4	0.89	0.2611	0.2998	0.2597	100%
4	9	0.90	0.2611	0.3652	0.2585	100%
3	1	0.93	0.2611	0.2280	0.2577	0%
1	7	0.89	0.2611	0.3098	0.2278	100%
4	6	0.89	0.2611	0.3652	0.2222	100%
11	4	0.97	0.2605	0.1798	0.2597	0%
14	9	0.90	0.2611	0.3985	0.2585	100%
14	2	0.88	0.2611	0.3985	0.1529	100%
11	7	0.96	0.2611	0.1798	0.2278	0%
14	6	0.89	0.2611	0.3985	0.2222	100%
8	8	0.89	0.2611	0.4708	0.2072	100%
13	1	0.90	0.2611	0.3738	0.2577	100%
8	7	0.89	0.2611	0.4708	0.2278	100%
1	1	0.90	0.2611	0.3098	0.2577	100%
6	9	0.90	0.2611	0.3099	0.2585	100%
6	1	0.90	0.2611	0.3099	0.2577	100%
2	6	0.89	0.2645	0.2625	0.2222	0%
1	7	0.89	0.2611	0.3098	0.2278	100%
3	2	0.92	0.2611	0.2280	0.1529	0%
4	1	0.90	0.2611	0.3652	0.2577	100%
6	5	0.89	0.2611	0.3099	0.2174	100%
8	7	0.89	0.2611	0.4708	0.2278	100%
7	2	0.88	0.2611	0.2998	0.1529	100%
14	2	0.88	0.2611	0.3985	0.1529	100%
6	4	0.90	0.2605	0.3099	0.2597	100%
		0.90±0.02				80%

**Table 4:** Influence of mean normalization on FuzzyEn. Parameters *m* and *r* were optimized again, achieving the best configuration for the same *r* values, but with m=2 instead of m=3 in this case.

Metric	*m*	*r*	*n*	*N*	AUC
FuzzyEn*	2	0.24	0.625	1728	0.83
FuzzyEn*	2	0.15	1	1728	0.79

**Table 5:** AUC results obtained for the analysis of the influence of missing samples on the classification capability.

Metric	0%	2.5%	5%	7.5%	10%
SampEn	0.90	0.83±0.01	0.77±0.02	0.75±0.01	0.73±0.02
FuzzyEn	0.82	0.80±0.01	0.76±0.01	0.75±0.04	0.74±0.02

**Table 6:** AUC results obtained for the analysis of the influence of reading saturation on the classification capability.

Metric	6	12	18	24	30	36	42	48	54	60
SampEn	0.82±0.0025	0.80±0.0050	0.79±0.0076	0.78±0.0031	0.80±0.0031	0.75±0.0152	0.73±0.0107	0.71±0.0165	0.72±0.0120	0.72±0.0355
FuzzyEn	0.77±0.0038	0.75±0.0025	0.76±0.0171	0.72±0.0076	0.73±0.0247	0.73±0.0311	0.70±0.0088	0.70±0.0184	0.69±0.0114	0.69±0209

**Table 7:** AUC performance of SampEn and FuzzyEn on a day-by-day basis. Analysis using 3-day time windows shifted 0 days up to 3 days to visualize the effect of the location of the data. Performance varies depending on the specific epoch being processed, with a clear trend towards better classification using the later epochs and a significant decay in performance at the beginning of the records.

Shift	AUC (SampEn)	AUC (FuzzyEn)
0	0.79 (p=0.0697)	0.69 (p=0.0991)
1	0.84 (p=0.0052)	0.83 (p=0.0087)
2	0.85 (p=0.0066)	0.77 (p=0.0290)
3	0.93 (p=0.0007)	0.81 (p=0.0109)
